# Stroke recovery beyond initial severity: the complementary roles of brain structure and brain function in acute stroke

**DOI:** 10.1093/braincomms/fcag355

**Published:** 2026-09-16

**Authors:** Catharina Zich, Lydia C Mardell, Andrew J Quinn, Sven Bestmann, Nick S Ward

**Affiliations:** Department of Clinical and Movement Neuroscience, UCL Queen Square Institute of Neurology, London WC1N3BG, United Kingdom; Wellcome Centre for Integrative Neuroimaging, FMRIB, Nuffield Department of Clinical Neurosciences, University of Oxford, Oxford OX39DU, United Kingdom; Medical Research Council Brain Network Dynamics Unit, University of Oxford, Oxford OX13TH, United Kingdom; Department of Clinical and Movement Neuroscience, UCL Queen Square Institute of Neurology, London WC1N3BG, United Kingdom; Centre for Human Brain Health, School of Psychology, University of Birmingham, Birmingham B152TT, United Kingdom; Department of Clinical and Movement Neuroscience, UCL Queen Square Institute of Neurology, London WC1N3BG, United Kingdom; Center for Clinical Neuroscience, Hospital Los Madroños, Brunete, Madrid 28690, Spain; Department of Imaging Neuroscience, Wellcome Centre for Human Neuroimaging, UCL Queen Square Institute of Neurology, London WC1N3AR, United Kingdom; Department of Clinical and Movement Neuroscience, UCL Queen Square Institute of Neurology, London WC1N3BG, United Kingdom

**Keywords:** acute stroke, mobile EEG, MRI, initial impairment, recovery

## Abstract

Stroke frequently results in long-term impairments, limiting independence and quality of life. Accurate prediction of recovery trajectories is essential for personalizing rehabilitation strategies. While structural brain metrics such as corticospinal tract (CST) integrity have been widely studied, they incompletely explain motor outcome variability. Functional brain activity, quantified by sensorimotor activity in the beta (β) frequency range, has emerged as a promising biomarker of motor system integrity and plasticity potential. This study examined 30 acute stroke survivors and 26 healthy controls, assessing how brain function and brain structure relate to initial severity and 6-month outcome accounting for initial severity, using clinical MRI/CT and electroencephalography during passive finger movement and rest. With respect to brain structure, grey and white matter damage were associated with initial severity, whereas no associations with 6-month outcome while accounting for initial severity were observed. In contrast, regarding brain function, β-activity in response to passive movement and resting state activity were related to 6-month outcome while accounting for initial severity, independent of initial severity. Multivariate regression analyses revealed that combining initial severity, structural information (CST damage) and brain function (sensorimotor β-activity) provided the most accurate model of both global and upper limb-specific 6-month outcome (*R*^2^ = 80.0% and 73.6%, respectively). These findings underscore the importance of integrating functional and structural neural markers for improved stroke outcome prediction.

## Teaser

Zich *et al*. report that in acute stroke, initial severity relates to structural brain damage, whereas 6-months outcome is linked to neural activity. Combining clinical, structural, and functional measures provides the most accurate model of outcomes, supporting the use of early neural biomarkers to guide rehabilitation strategies.

## Graphical Abstract

**Graphical Abstract:**
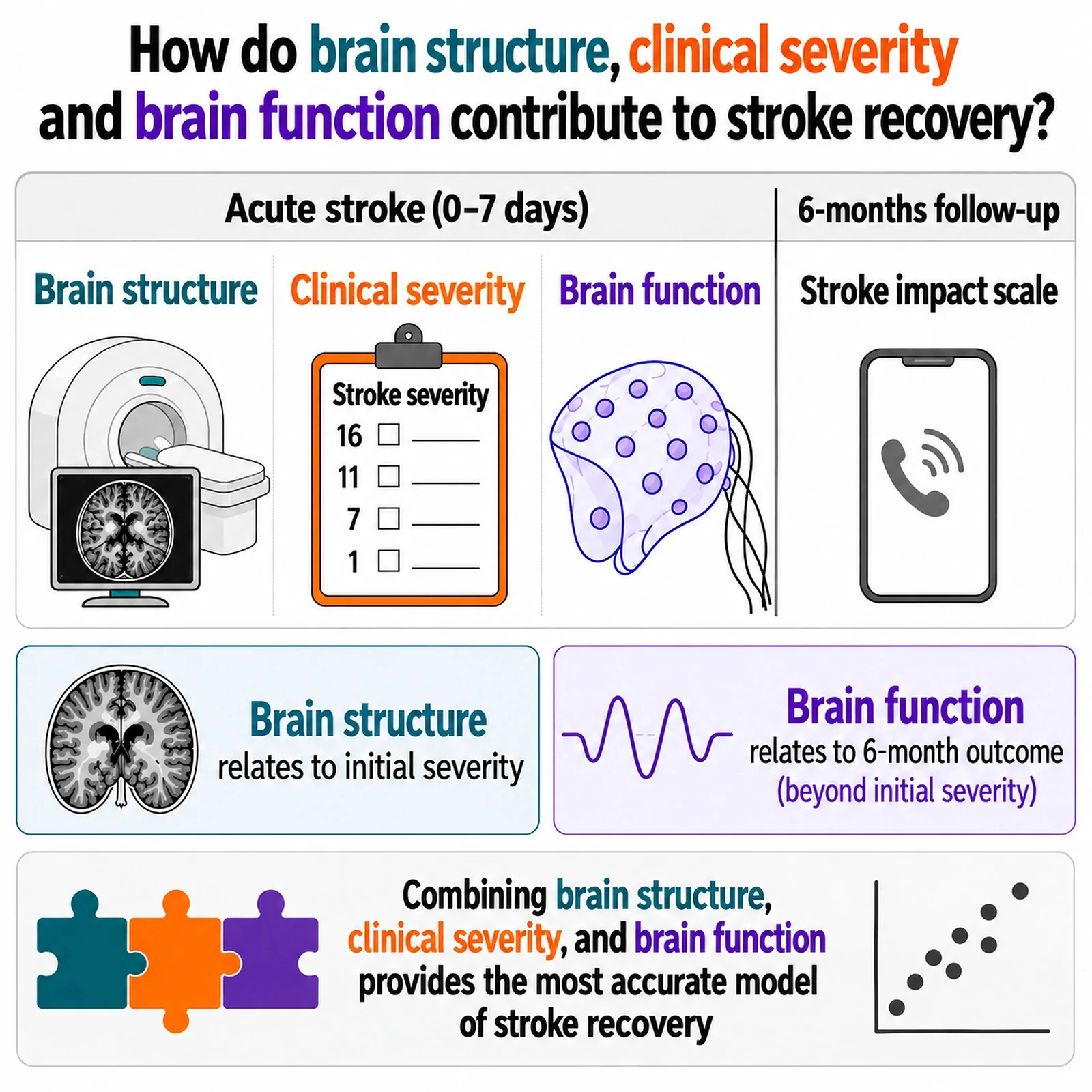

## Introduction

Stroke is a leading cause of long-term disability, with one in four people affected in their lifetime. Among survivors, about 25% remain moderately to severely disabled after 10 years.^1^ Improving long-term outcomes requires an understanding of the mechanisms of both initial impairment and subsequent recovery trajectories.^2^ While upper limb (UL) impairment is common and often limits daily independence and quality of life,^3^ stroke can affect multiple domains. Therefore, a comprehensive evaluation of both overall impairment and UL-specific outcomes is essential.

Long-term outcomes after stroke are related to initial impairment,^4^ which itself reflects the characteristics of structural brain damage. For example, the integrity of descending white matter (WM) pathways, most notably the corticospinal tract (CST), is associated with the severity of initial UL function/impairment in the acute stage after stroke [Action Research Arm Test (ARAT),^5,6^ Box and Block (BB),^7^ Fugl-Meyer Assessment for upper extremity (FM-UE),^8,9^ Nine-Hole Peg Test (NHPT),^7^ NIH Stroke Scale (NIHSS),^6,7,9^ grip strength^7^]. However, it remains the case that stroke survivors with the same level of initial UL impairment can have very different long-term UL outcomes, especially in those with more severe initial UL impairment/function (FM-UE,^10-12^ NHPT^13^). These differences in long-term UL outcomes can be partly explained by different levels of post-stroke CST integrity (ARAT,^5^ FM-UE,^14-16^ NIHSS^15^)^17^ but substantial unexplained variability in long-term UL outcomes remains. This raises the question of whether the mechanisms of initial impairment and the mechanisms of subsequent recovery are different and potentially independent.^2,13^ The International Stroke Recovery and Rehabilitation Alliance consensus paper suggested that the mechanisms of recovery might be more closely related to post-stroke brain function, highlighting several potential biomarkers, including cortical neural activity as measured with electroencephalography (EEG) or magnetoencephalography (MEG).^18^ Identifying brain function biomarkers of the recovery process itself, independent from the mechanisms of initial impairment would be an important finding since brain function is more amenable to therapeutic intervention for enhancing recovery than brain structure.^2^

Sensorimotor beta (β) activity (13–30 Hz) is a promising biomarker of motor function. Movement is accompanied by β-suppression (event-related desynchronization, ERD) reflecting sensorimotor cortex activation, followed by a rebound (event-related synchronization, ERS) linked to cortical inhibition or deactivation.^19-21^ This ERD-ERS pattern is robust and reproducible.^22,23^ β-activity is driven by GABA-A–mediated inhibitory interneuron activity^24-26^ and therefore reflects shifts in the cortical excitatory-inhibitory balance that could mediate experience-dependent plasticity.^2^ Indeed, changes in sensorimotor cortex β-activity have been linked to motor skill learning in healthy controls (HC)^27-31^ and stroke survivors,^32,33^ with stronger β-activity associated with faster adaptation and more efficient error correction during learning. Mechanistically, β-activity is thought to regulate the balance between maintaining the current motor state and facilitating changes in motor output, acting as a ‘status quo’ signal that stabilizes ongoing sensorimotor activity while allowing for trial-by-trial updating during learning.^28,29,31^ After stroke, β-activity can be reduced,^13,34-37^ potentially impairing these learning-related processes and limiting the ability to refine motor commands based on sensory feedback. Moreover movement-related low-frequency activity has been shown to track motor impairment (FM-UE) and recovery after (FM-UE) stroke,^38^ and high-frequency activity may provide additional mechanistic insights,^39^ although evidence in stroke populations is still limited. We focus on β-activity because of its mechanistic link to sensorimotor cortex excitability and its accessibility via EEG during passive or imagined movements, making it suitable for severely impaired patients. To evaluate whether post-stroke changes are limb-specific, task-dependent, or reflect more global alterations in cortical processing, EEG data are acquired during passive movement of both the affected and unaffected index fingers as well as during resting state.

In this study, we leveraged routinely collected stroke service data and bedside observations to evaluate how brain structure and function relate to global and UL-specific severity and 6-month outcome accounting for initial severity, with the goal of informing clinically feasible prognostic approaches. We hypothesized that (i) initial severity is primarily driven by structural damage to key grey and WM, reflecting the extent of tissue loss; (ii) 6-month outcome accounting for initial severity depends more on preserved or adaptive neural activity, reflecting the capacity for cortical plasticity; and (iii) measures of brain function would account for some variability in 6-month outcome, beyond initial severity and structural damage, highlighting early neural activity as a clinically actionable marker of recovery potential.

## Materials and methods

This study included a group comparison of HC and stroke patients, alongside analyses within the stroke cohort to examine associations with severity (NIHSS) and 6-month outcome (SIS). NIHSS and task-based EEG during passive UL movements, as well as resting-state EEG, were acquired within the first week post-stroke, together with routine structural magnetic resonance imaging (MRI) and computed tomography (CT).

## Experimental design

### Ethical approval

The Research Ethics Committee of the University College London and the NHS Research Ethics Committee (London—Surrey Research Ethics Committee) approved the study protocol (20/LO/0520), and all subjects provided written informed consent.

### Subjects

Acute stroke survivors (1–7 days,^40^) with first-ever stroke and motor deficits (as recorded in the clinical admission notes), aged 18 years and older, based at the Hyper-acute stroke unit and acute stroke unit at The National Hospital for Neurology and Neurosurgery (NHNN), Queen Square. Exclusion criteria comprised: medically unstable, requiring neurosurgical intervention, severe language/cognitive deficits preventing consent (determined by clinical judgment in collaboration with physiotherapists, occupational therapists, and speech-language therapists), inability to understand English, or sign their name, presence of other motor disorders (e.g. Parkinson’s disease), or inability to achieve proper EEG electrode placement due to hair or head coverings. HC were recruited via advertisements and reported no current psychiatric or neurological disorders. They were recruited to approximately match the age distribution of the stroke cohort.

### Clinical data

Initial global severity was measured using the NIHSS, with UL-specific severity based on its UL sub-scores (item 5 ‘Motor arm’). The NIHSS was selected as it is a standardized and widely used measure of neurological severity that is routinely collected in clinical practice, facilitating integration with standard clinical and neuroimaging data and supporting broader applicability across studies and cohorts. The NIHSS was administered at admission (M = 6.6, SD = 6.0; range: 0–20 points).

Global 6-month outcome was assessed using the total score of the Stroke Impact Scale (SIS) at 6 months post-stroke (M = 6.2 months, SD = 0.3; range: 5.8–7.1 months). The SIS was used as a multidimensional measure of post-stroke recovery, assessing physical, cognitive, emotional, and social domains; it has demonstrated strong structural and construct validity, convergent validity with established outcome measures,^41,42^ and can be administered either in person, by phone, or using pen-and-paper formats, facilitating flexible data collection across settings. Additionally, the SIS is widely collected in clinical stroke services, making it highly relevant to current clinical practice. UL-specific 6-month outcome was measured by the UL subscale of the SIS (average of section ‘1 Physical’ and ‘7 Affected Arm’). The SIS was collected via online questionnaire, paper form, or phone interview, depending on patient preference. Analyses of global and UL-specific 6-month outcome were adjusted for initial global or UL-specific initial severity by including the NIHSS total score or the NIHSS UL sub-score, respectively, as covariates in the regression models.

### EEG data

#### EEG data acquisition

EEG data were recorded with sintered Ag/AgCl electrodes from 30 scalp sites using electrode caps (Easycap, Herrsching, Germany), with AFz as the ground and FCz as the reference. Data were acquired with an amplitude resolution of 0.1 μV and a sampling rate of 500 Hz, using online analogue filter settings of 0.02 to 250 Hz, via a wireless amplifier (SmartingPRO, mBrainTrain, Belgrade, Serbia) attached to the back of the EEG cap. Data were transmitted via Bluetooth 5.0 to a laptop (Dell Latitude 7390) via a Bluetooth dongle (VCK dongle). No data were lost during transmission. Impedances were checked at the beginning (Smarting Streamer software v4.1; mBrainTrain, Belgrade, Serbia) and recorded throughout the experiment with a sampling rate of 125 Hz. Impedances were on average 17.4 kΩ (SD = 9.4 kΩ) in stroke survivors and 15.5 kΩ (SD = 5.9 kΩ) in HC.

Patient data were recorded on five active hospital wards of the hyper-acute stroke unit and acute stroke unit at The National Hospital for Neurology and Neurosurgery (NHNN), Queen Square. Stroke survivors were, depending on their clinical condition and preference, either in a sitting (*N* = 13) or supine position (*N* = 17). Data from HCs were recorded in a side room of the same building while they were seated.

#### Passive movement task and resting state

Data were collected during passive movement of the left and right index fingers using a block design. We used a passive movement as this provides access to sensorimotor β-activity, unconfounded by residual movement. The subject’s index finger was positioned above a keyboard key (Ecarke OSU Keypad 4-Key Gaming Keyboard) and held in place using a Velcro sleeve. The experimenter moved the key down and immediately up roughly every 5 s (Stroke survivors: M = 5.1 s, SD = 0.2 s; HC M = 5.1 s, SD = 0.1 s) for 6 min (Stroke survivors: M = 67.3, SD = 3.1, range 61–74 trials; HC M = 67.9, SD = 1.4, range 64–70 trials). Participants were instructed to relax, not to move their head or fingers, and not to pay attention to the stimuli.

Moreover, data were collected during rest with eyes open (Stroke survivors: M = 7.0 min, SD = 0.4 min; HC M = 7.0 min, SD = 0.1 min).

#### EEG data pre-processing

EEG data were preprocessed with the EEGLAB toolbox version v2021.1^43^ for MATLAB (version 2022a; MathWorks, Natick, MA, USA). First, to suppress high frequencies, data were low-pass filtered (35 Hz, FIR, hamming window, filter order: 166). Data were downsampled to 250 Hz to reduce computational demands and disk space consumed, and high-pass filtered at (1 Hz, FIR, hamming window, filter order: 826) to suppress very slow trends in the data. Edge artefacts were removed by discarding the first and last 5 s of each recording. Identification of improbable channels was conducted using the EEGLAB extension trimOutlier (https://sccn.ucsd.edu/wiki/EEGLAB_Extensions) with an upper and lower boundary of two standard deviations of the mean standard deviation across all channels (Stroke survivors: M = 1.6, SD = 0.6, range 0–3 channels; HC M = 0.5, SD = 0.9, range 0–3 channels). Channels exceeding this threshold were replaced by spherical interpolation. Two copies of the dataset were created. One copy was cleaned using Artifact Subspace Reconstruction (ASR, as implemented in EEGLAB plugin clean_rawdata(),^44-46^) aiming to remove non-stereotypical artefacts to improve Independent Components Analysis (ICA). We used temporal ICA across the channels using the runica algorithm^47^ to estimate 30 independent components. Components reflecting line noise, eye, muscle and heart activity were identified using the EEGLAB extension ICLabel^48^ and controlled by visual inspection. Components flagged as artefactual (Stroke survivors: M = 6, SD = 2.9, range 4–10 components; HC M = 5.9, SD = 2.3, range 4–11 components) were regressed out of the second copy created after spherical interpolation.

For the resting state data, the power spectral density (PSD) estimate was computed with a 2 s window with a 1 s overlap. The spectra were normalized to represent relative power by dividing each bin in the spectrum by the total power across all bins. For each participant, this 2-dimensional array of the spectrum for each channel (frequencies × sensors) was computed and used in the group analysis.

Task data were segmented from −1.5 to 3.5 s relative to the button press. Artefactual epochs as indicated by the joint probability (EEGLAB function pop_jointprob.m, SD = 3) were discarded from further analyses (Stroke survivors: M = 11.1%, SD = 3.4%, range 7.3–18.9%; HC *M* = 10.3%, SD = 3.4%, range 5.9–24.8%). Time-frequency representations of individual trials were calculated using the Fieldtrip functions (www.fieldtriptoolbox.org;^49^) integrated in SPM12 using Morlet wavelet analysis with a wavelet width of 9. Power values were baseline corrected (−1 to −0.2 s). Data were averaged across the β−(13–30 Hz) frequency range, resulting in two-dimensional data (time × space). Stroke survivor data were grouped as affected and unaffected hands, while healthy control data were grouped as non-dominant and dominant hand. Data from patients with left-hemisphere lesions were mirrored, aligning the right hemisphere with the affected side and the left hemisphere with the unaffected side, and the left and right hands with the affected and unaffected hands, respectively.

### MRI and CT data

#### MRI and CT data acquisition

CT (*N* = 28) and MRI (*N* = 27) data were acquired as part of the hospital routine. T1 and T2 sequences (TR = 4000 ms; TE = 101 ms; voxel size = 1.59 × 1.59 × 6.5 mm^3^; matrix = 172 × 172, 23 slices; flip angle = 90 deg) were obtained using a 1.5T Siemens Avanto MRI scanner (Siemens Healthineers, Germany). CT sequences (matrix = 512 × 512; with 34, 108, 112, 217 slices) were obtained using a Siemens GoAll or Siemens XCite CT scanner.

#### Lesion mapping and quantification of lesion volume, GM damage and WM damage

Stroke lesions were demarcated using the semi-automated segmentation algorithm *Clusterize* (http://www.medizin.uni-tuebingen.de/kinder/en/research/neuroimaging/software/) applied to axial T2, T1 or CT images. Agreement between a manual segmentation and the semi-automated lesion maps obtained with *Clusterize* has been previously demonstrated.^50-52^ The resulting lesions were manually verified and, if necessary, manually corrected. Lesion maps were smoothed using a 2 mm full-width half maximum (FWHM) Gaussian kernel. Lesions were normalized to standard MNI space using cost-function masking (CFM) to avoid distortions,^53^ and left hemispheric lesions were flipped.

We calculated the lesion volume, GM damage and WM damage using the *Lesion Quantification Toolkit* (https://github.com/cathazi/Zich_Copy_LQT.git).^54^ Grey matter (GM) damage is quantified as the percentage of voxels within each region of the automated anatomical labelling (AAL) atlas^55^ that overlapped with the lesion. We obtained GM damage for each region separately as well as total GM damage (i.e. mean across all regions). WM damage is quantified as the percentage of streamlines of the HCP842 tractography atlas (Yeh *et al*. 2018)^56^ that intersect the lesion. We obtained WM damage for each tract separately as well as total WM damage (i.e. mean across all tracts).

#### Statistical analysis

Brain functional data were analysed using a General Linear Model (GLM) framework as implemented in *glmtools* (https://pypi.org/project/glmtools/;^57^). Two GLMs (task, rest) were constructed to compare group averages, each with three regressors: two categorical predictors using dummy coding for participant group (healthy/acute), and one continuous covariate representing z-transformed participant age. Parameters were estimated using the Moore-Penrose Pseudo-Inverse for each frequency–sensor pair in the resting-state data, resulting in a spectrum of parameters. For the task data, parameters were estimated for each time–sensor pair, yielding a time course of parameters. A contrast of parameter estimates was defined to test for differences in the parameters between the healthy and acute groups. The resulting contrast estimates (COPEs) and their associated standard errors are used to compute a *t*-test for this contrast.

Four additional parametric models were constructed to assess correlations with both initial severity and 6-month outcome, while accounting for initial severity, in the patient group for both task and rest data. Specifically, two GLMs were developed to examine correlations with initial severity (NIHSS) for task and rest data. Each model included one constant regressor and one parametric regressor, comprising the *z*-transformed NIHSS values for each participant. Additionally, two further models were constructed to assess 6-month outcome (SIS), while controlling for initial severity (NIHSS), again for both task and rest data. These models contained a single constant regressor and two parametric regressors containing the *z*-transformed values for SIS and NIHSS for each participant. Parameters were estimated using the Moore-Penrose Pseudo-Inverse and a contrast of parameter estimates defined to test for differences between the parameters of each variable and zero. The resulting contrast estimates (COPEs) and their associated standard errors are used to compute a *t*-test for this contrast.

Null-hypothesis testing was conducted using cluster based non-parametric permutation tests.^58,59^ This approach makes minimal assumptions, addresses the issue of multiple comparisons, and is suitable when parametric assumptions are not met.^59^ Row-shuffle permutations were used to compare subgroup differences and test relationships between variables, while sign-flipping permutations were used to test whether a measurement deviated from zero. A null distribution of cluster statistics was generated by permuting the data and calculating the sum of *t*-values within the largest contiguous cluster exceeding a cluster-forming threshold of *t* = 3.5. Observed cluster statistics were compared with this null distribution and considered significant if they exceeded a predefined critical value. In this study, the null distribution was constructed using 5000 permutations, with the 95th percentile used as the significance threshold. Multiple comparisons were controlled for within each statistical map using a cluster-based permutation approach. No formal correction was applied across the different models.

When correlations were assessed, Spearman’s rank correlation was used due to non-normality of the data, and confidence intervals were computed using permutation bootstrapping with 5000 iterations.

Stepwise regression analysis was conducted using R (version 4.0.2) and independent variables were standardized using *z*-transformation. First, a linear regression with initial severity as independent variable and 6-month outcome as dependent variable was performed. This initial model was compared with models in which brain structure and brain function were added separately as independent variable. These models were, in turn, compared with a model comprising initial severity, brain structure, and brain function as independent variables. The results were confirmed by forward stepwise linear regression. To this end, at each step, independent variables were included when *P* < 0.15 (Wald test) and removed when *P* ≥ 0.15 (Wald test). Independent variables showing high collinearity (variance inflation factor (VIF) > 2.5) were re-assessed. *R*^2^ and RMSE were used to quantify the models’ overall ability to model 6-month outcome. Moreover, the relationship between each independent variable and the dependent variable was described by the regression coefficients. Sensitivity analyses were conducted on the final model to assess whether demographic and lesion-related variables influenced the observed associations. Age, lesion side, and time post-stroke were each entered individually as additional covariates in the final multivariable regression models. Covariates were added one at a time to minimize overfitting and model instability given the sample size.

Given the available sample sizes, post hoc sensitivity analyses indicated 80% power (*α* = 0.05) to detect correlations or standardized regression coefficients of approximately |*r*|/|*β*| ≥ 0.5 (*N* = 30), and medium-to-large group differences (Cohen’s *d* ≈ 0.75) for between-group comparisons (*N* = 30 versus *N* = 26).

## Results

### Participants

375 acute stroke survivors were screened within 7 days of stroke, of which 32 were recruited (Fig. 1A). Two datasets were excluded: one participant aborted the session, and one dataset was corrupted by an unspecific artefact, resulting in 30 datasets for the main analysis (4 females, age: M = 64.7; SD = 13.5; range 30.3–94.4 years, Table 1, Fig. 1B_i_). At the time of the EEG the time since stroke was M = 3.5; SD = 1.8; range 1–7 days (Fig. 1B_ii_). Due to constraints related to clinical workflow on the acute ward, resting state data could not be obtained in nine stroke survivors. Three stroke survivors did not complete the SIS follow-up. EEG data during passive movement and rest were recorded from 26 HC (9 females, age: M = 56.5; SD = 11.1; range 37.7–85.2 years, Fig. 1B_i_) in an office room within the same hospital building.

**Figure 1:**
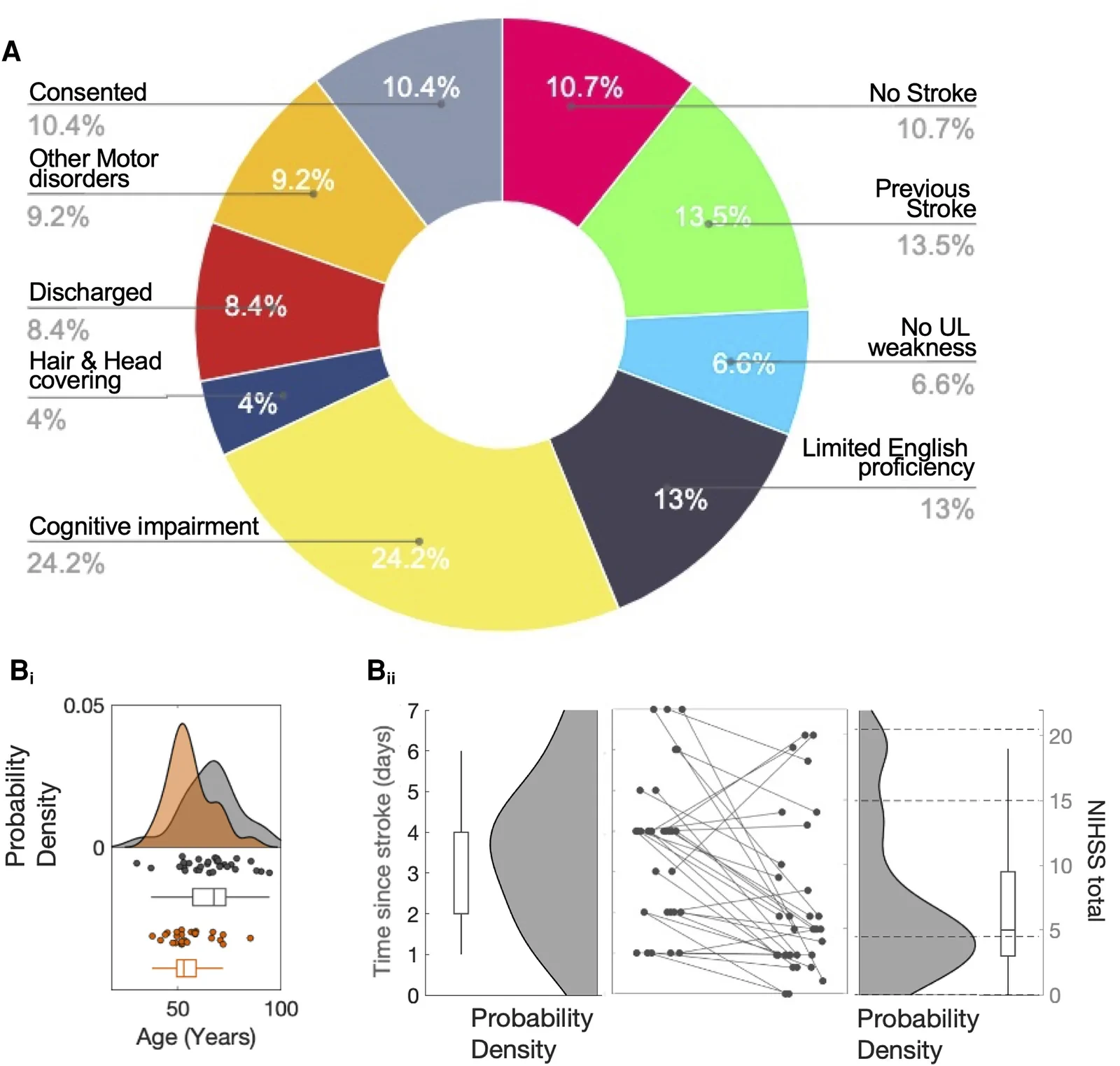
**Recruitment chart and selected demographics.** (**A**) Inclusion and exclusion criteria for 375 screened patients. (**B_i_**) Age for HC (red, *N* = 26) and stroke survivors (grey, *N* = 32). Data are shown as probability function, individual subjects, and boxplot [median, interquartile range (25th–75th percentiles), whiskers extending to ±1.5× IQR]. (**B_ii_**) Time since the stroke and NIHSS total for stroke survivors. Data are shown as boxplot [median, interquartile range (25th–75th percentiles), whiskers extending to ±1.5× IQR], probability function, and individual subjects.

**Table 1:** Clinical details of the stroke survivors

ID	Age	Days	Type	Affected hand	Location/description	NIHSS
1	66–70	1	I	R	Pons	3
2	71–75	1	H	L	BG	3
3	66–70	1	I	R	Precentral gyrus	0
4	36–40	6	H	R	Pons	6
5	51–55	2	I	R	Striato-capsular	4
6	51–55	7	H	L	Parietoccipital	1
7	76–80	2	I	L	Precentral gyrus	2
8	66–70	4	I	R	Corona Radiata	3
9	66–70	2	I	R	PCA	3
10	86–90	2	I	L	Precentral gyrus	2
11	26–30	4	I	R	Precentral gyrus	6
12	51–55	2	I	L	PICA	0
13	56–60	5	I	L	BG, Corona Radiata	3
14	71–75	4	I	L	Perirolandic region, corona radiata	9
15	51–55	4	H	R	BG	13
16	56–60	4	I	L	Posterior lentiform	5
17	66–70	4	I	L	Hemipons	5
18	71–75	3	I	R	M1, BG	20
19	61–65	3	I	L	MCA, M1	2
20	61–65	4	I	L		19
21	61–65	4	I	L	BG	6
22	81–85	2	I	L	Frontal & parietal lobe	5
23	66–70	4	H	L	temporo-occipital lobe	3
24	91–95	4	I	L	MCA, posterior-inferior frontal lobe & insula	6
25	66–70	1	I	R	MCA, S2	5
26	51–55	7	H	L	BG	18
27	61–65	7	I	L	L PICA, R PCA	5
28	51–55	6	H	R	Thalamus	14
29	66–70	2	I	R	Striatocapsular	8
30	56–60	4	I	L	Large MCA	20
Stroke (*N* = 30)	64.7 (30.3–94.4)	3.5 (1–7)	23I/7H	18L/12R		6.6 (0–20)
Controls (*N* = 26)	56.5 (37.7–85.2)	-	-	-	-	-

F, female; H, haemorrhagic; I, ischemic; L, left; M, male; NIHSS, National Institutes of Health Stroke Scale; R, right.

### Stroke-related changes in brain structure and function

First, we examined stroke-related changes in brain structure. *Lesion volume:* Stroke-related damage to brain structure particularly involved subcortical GM and WM structures in the middle cerebral artery (MCA) territory (Fig. 2A_i_) (mean lesion volume = 16.92 mL, SD = 18.29 mL, range 1.01–63.56 mL, Fig. 2A_ii_). *Grey matter*: GM damage most commonly involved the pallidum, putamen and amygdala (Fig. 2B_i_, Table 2) (mean total GM damage = 0.97%, SD = 1.20%, range 0.03–4.69%, Fig. 2B_ii_). *White matter*: WM damage most commonly involved corticospinal, parietopontine, and frontopontine tracts (Fig. 2C_i_, Table 3) (mean total WM damage = 7.78%, SD = 7.36%, range 0.34–34.90%, Fig. 2C_ii_).

**Figure 2:**
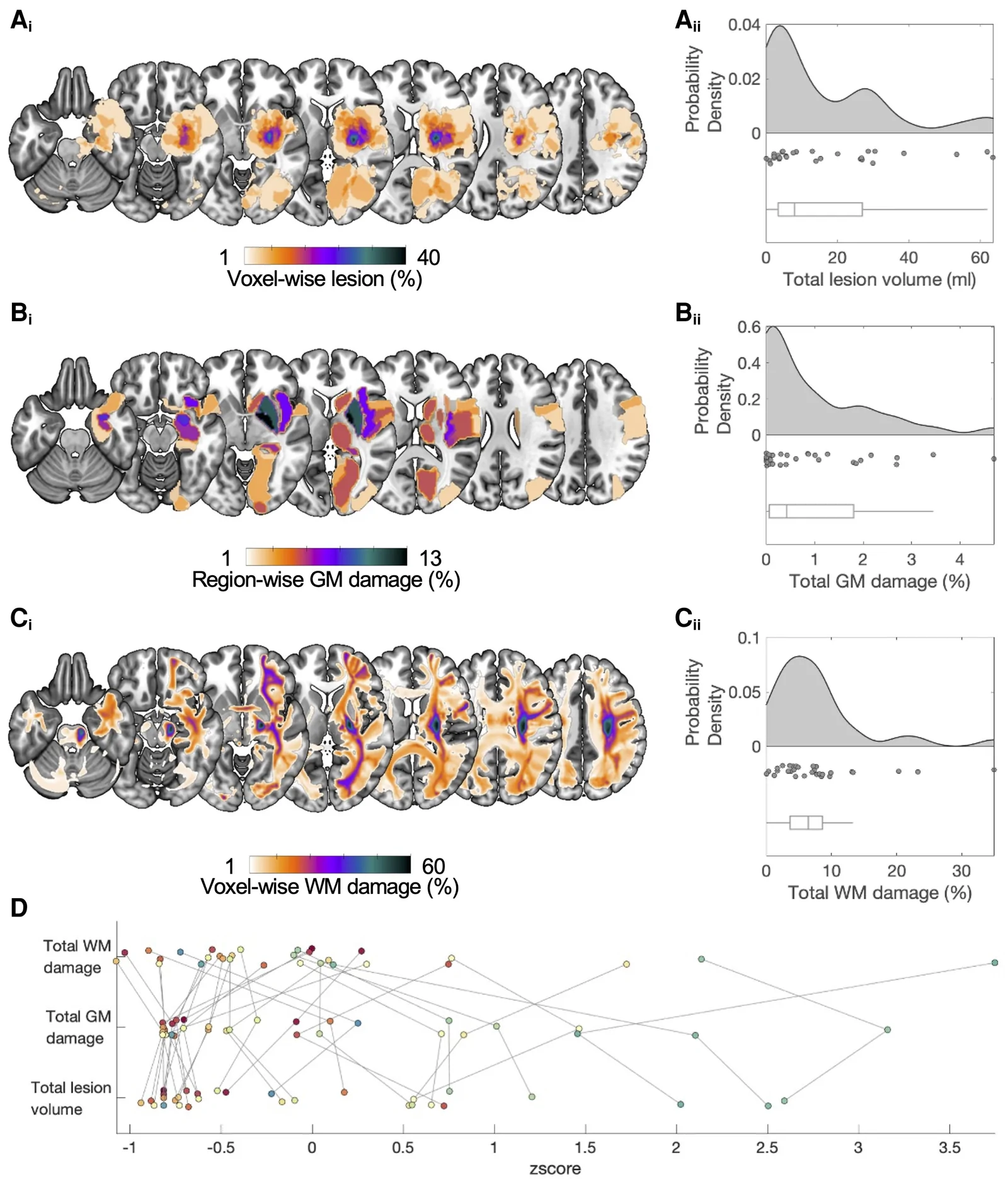
**Lesion-related alterations in brain structure. (A_i_) Group voxel-wise lesion map overlaid on an MNI template. Left hemispheric lesions were flipped.** (**A_ii_**) Total lesion volume as probability function, individual subjects, and boxplot [median, interquartile range (25th–75th percentiles), whiskers extending to ±1.5× IQR]. (B_i_) Group region-wise map of GM damage overlaid on an MNI template. Left hemispheric lesions were flipped. GM damage most commonly involved the pallidum, putamen and amygdala. (**B_ii_**) Total GM damage as probability function, individual subjects, and boxplot [median, interquartile range (25th–75th percentiles), whiskers extending to ±1.5× IQR]. (**C_i_**) Group voxel-wise map of WM damage overlaid on an MNI template. Left hemispheric lesions were flipped. WM damage most commonly involved corticospinal, parietopontine, and frontopontine tracts. (**C_ii_**) Total WM damage as probability function, individual subjects, and boxplot [median, interquartile range (25th–75th percentiles), whiskers extending to ±1.5× IQR]. (**D)** Normalized (*z* score) total lesion volume, total GM damage, and total WM damage for each stroke survivor.

**Table 2:** Details of GM damage for the 10 most damaged regions

Rank	Label	No of patients with damage	M (%) ± SD (%)	Range (%)
1	Lenticular nucleus, Pallidum	10/30	12.70 ± 26.40	0–98.04
2	Lenticular nucleus, Putamen	16/30	11.39 ± 20.29	0–82.07
3	Amygdala	8/30	8.49 ± 21.18	0–99.95
4	Insula	8/30	7.38 ± 15.76	0–63.21
5	Hippocampus	9/30	6.29 ± 15.88	0–56.99
6	Heschl’s gyrus	6/30	5.53 ± 16.58	0–72.39
7	Caudate nucleus	12/30	5.36 ± 12.18	0–58.51
8	Thalamus	12/30	5.24 ± 17.33	0–92.08
9	Calcarine fissure and surrounding cortex	5/30	4.90 ± 15.53	0–73.94
10	Rolandic operculum	8/30	4.86 ± 13.44	0–52.70

**Table 3:** Details of WM damage for the 10 most damaged tracts

Rank	Label	No of patients with damage	M (%) ± SD (%)	Range (%)
1	Corticospinal tract	25/30	47.83 ± 39.04	0–100
2	Parietopontine tract	26/30	39.84 ± 38.17	0–100
3	Frontopontine tract	24/30	31.08 ± 37.96	0–100
4	Inferior fronto occipital fasciculus	16/30	30.36 ± 40.74	0–100
5	Arcuate Fasciculus	14/30	28.52 ± 42.13	0–100
6	Temporopontine tract	15/30	27.11 ± 40.05	0–100
7	Anterior commissure	14/30	26.34 ± 39.73	0–99.91
8	Extreme capsule	14/30	25.74 ± 39.75	0–100
9	Occipitopontine tract	16/30	25.70 ± 36.39	0–100
10	Superior longitudinal fasciculus	13/30	19.53 ± 27.64	0–82.14

Next, we investigated stroke-related differences in brain function. *Passive movement—affected hand*: Cluster-based analysis revealed a significant β-ERD cluster with lower ERD (less negative) in stroke survivors than HC, extending from −0.30 to 0.35 s and involving 16 bilateral parietal and occipital channels (*threshold*_cluster_forming_ = 3.5, *p*_cluster_threshold_ = 0.01, *rank*_cluster_ = 1, *T*_cluster___sum_ = −360.14, *T*_cluster_max_ = −5.26, *p*_cluster_ < 0.001, β-ERD difference range = −62.75 to −5.89, mean difference = −36.51, Fig. 3A_iii_). β-ERS (1.00 to 2.20 s relative to passive movement) was lower (less positive) in stroke survivors compared with HC in 5 channels across the contralateral (ipsilesional) sensorimotor area (*threshold*_cluster_forming_ = 3.5, *p*_cluster_threshold_ = 0.01, *rank*_cluster_ = 2, *T*_cluster___sum_ = 268.46, *T*_cluster_max_ = 4.54, *p*_cluster_ < 0.001, β-ERS difference range = 20.96 to 66.70, mean difference = 43.58, Fig. 3A_iii_). *Passive movement—unaffected hand*: No group difference was observed for β-ERD (SI Fig. 1). β-ERS was reduced (less positive) in stroke survivors compared with HC above contralateral (contralesional) and ipsilateral (ipsilesional) sensorimotor areas (SI Fig. 1A, SI Table 1). Stroke-related reductions in β-ERS (less positive) are not specific to the affected hand. *Resting state:* There were no significant differences between HC and stroke survivors in resting state power at any frequency and any channel (*threshold*_cluster_forming_ = 3.5, *p*_cluster_threshold_ = 0.01, Fig. 3B).

**Figure 3:**
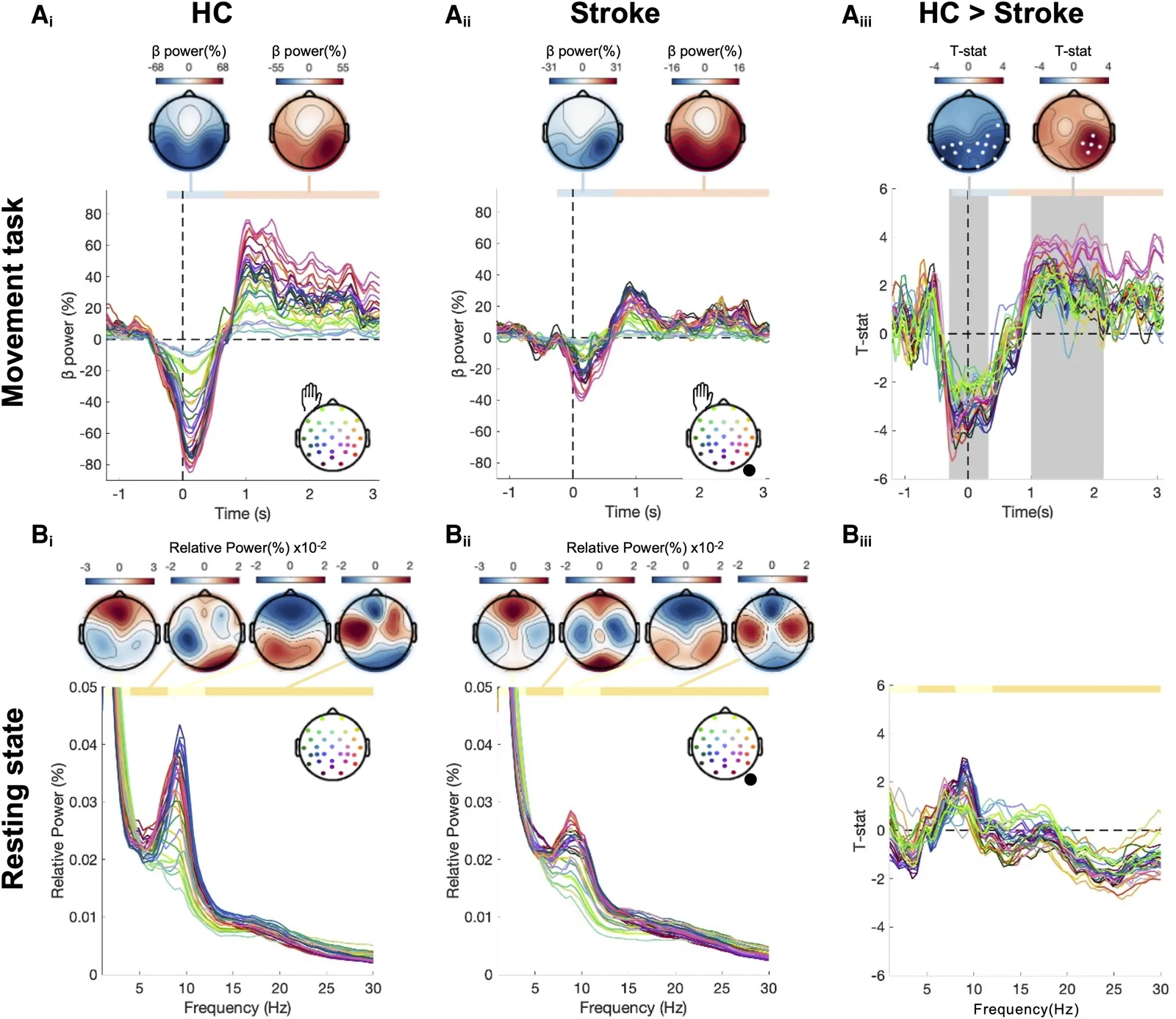
**Brain function in acute stroke relative to HC. (Ai) β-power during passive movement for HC of the non-dominant hand.** Mean power estimates as a function of time and space. The inlay illustrates the channel location colour coding and the hand that was moved (non-dominant hand). ERD and ERS time windows are highlighted in blue and red accordingly. Topographies show the spatial pattern of β-power averaged over ERD and ERS time windows. (**Aii**) Same as (Ai) for passive movement of the affected hand in stroke survivors. The inlay illustrates the channel location colour coding, the lesioned hemisphere (black circle) and the hand that was moved (here affected hand). (**Aiii**) *t*-values as a function of time and space for the contrast between HC (*N* = 26) and acute stroke survivors (*N* = 30). Statistical significance is assessed by time × channel cluster permutation testing (*threshold*_cluster_forming_ = 3, *p*_cluster_threshold_ = 0.01). Significant times are indicated in by the grey area. Significant channels are highlighted in white in the topography. (**Bi**) Resting state power for HC. Mean power estimates as a function of frequency and space. The inlay illustrates the channel location colour coding. δ−, θ−, α−, β-frequency bands are highlighted in different shades of yellow. Topographies on the top show the spatial pattern of power averaged over each frequency band. (**Bii**) Same as (Bi) for stroke survivors. The inlay illustrates the channel location colour coding, the lesioned hemisphere (black circle). (**Biii**) *t*-values as a function of frequency and space for the contrast between HC (*N* = 26) and acute stroke survivors (*N* = 21). Statistical significance is assessed by frequency × channel cluster permutation testing *threshold*_cluster_forming_ = 3, *p*_cluster_threshold_ = 0.01). No significant difference was found.

Next, we wanted to test whether stroke-related changes in brain structure and function are related. We found that stronger β-ERS (more positive) induced by passive movement of the affected hand was related to lower lesion volume [*rho*(*28*) = −0.50, 95% bootstrap CI (−0.72, −0.17), *P* = 0.006] and lower total GM damage [*rho*(*28*) = −0.61, 95% bootstrap CI (−0.80, −0.31), *P* < 0.001], but not with total WM damage [*rho*(*28*) = −0.35, 95% bootstrap CI (−0.65, 0.33), *P* = 0.056]. Interestingly, a similar relationship was found between for β-ERS induced by passive movement of the unaffected hand with lesion volume [*rho*(*28*) = −0.38, 95% bootstrap CI (−0.63, −0.01), *P* = 0.039] and total GM damage [*rho*(*28*) = −0.46, 95% bootstrap CI (−0.72, −0.08), *P* = 0.011], but again not with total WM damage [*rho*(*28*) = −0.07, 95% bootstrap CI (−0.44, 0.34), *P* = 0.693]. No significant relationships were observed between passive movements induced β-ERD (for either hand) and brain structure metrics (all *p*’s > 0.1).

### Initial severity relates to brain structure but not brain function in acute stroke

We tested whether initial global or UL-specific severity (NIHSS) was related to structural brain damage. Larger lesion volume was associated with greater initial global severity [*rho*(*28*) = 0.39, *P* < 0.05], but not with initial UL-specific severity (*P* > 0.1). Greater global initial severity was also associated with greater total GM damage [*rho*(*28*) = 0.40, 95% bootstrap CI (0.06, 0.68), *P* < 0.05, Fig. 4Aii], while greater initial UL-specific severity was associated with to greater damage to the pallidum [*rho*(*28*) = 0.49, 95% bootstrap CI (0.10, 0.72), *P* < 0.05], putamen [*rho*(*28*) = 0.48, 95% bootstrap CI (0.18, 0.75), *P* < 0.01], and caudate [*rho*(*28*) = 0.47, 95% bootstrap CI (0.15, 0.74), *P* < 0.01, Fig. 4Bii]. Greater initial global severity was related to greater damage to the corticospinal [*rho*(*28*) = 0.40, 95% bootstrap CI (0.02, 0.67), *P* < 0.05], corticostriatal [*rho*(*28*) = 0.38, 95% bootstrap CI (−0.01, 0.69), *P* < 0.05] and frontopontine tracts [*rho*(*28*) = 0.44, 95% bootstrap CI (0.06, 0.74), *P* < 0.05, Fig. 4Aiii]. Greater initial UL-specific severity was associated with greater damage to the corticospinal [*rho*(*28*) = 0.49, 95% bootstrap CI (0.13, 0.75), *P* < 0.01], corticothalamic [*rho*(*28*) = 0.41, 95% bootstrap CI (0.03, 0.71), *P* < 0.05], corticostriatal [*rho*(*28*) = 0.45, 95% bootstrap CI (0.11, 0.72), *P* < 0.05], parietopontine [*rho*(*28*) = 0.51, 95% bootstrap CI (0.10, 0.79), *P* < 0.01], and frontopontine tracts [*rho*(*28*) = 0.50, 95% bootstrap CI (0.12, 0.78), *P* < 0.01, Fig. 4Biii].

**Figure 4:**
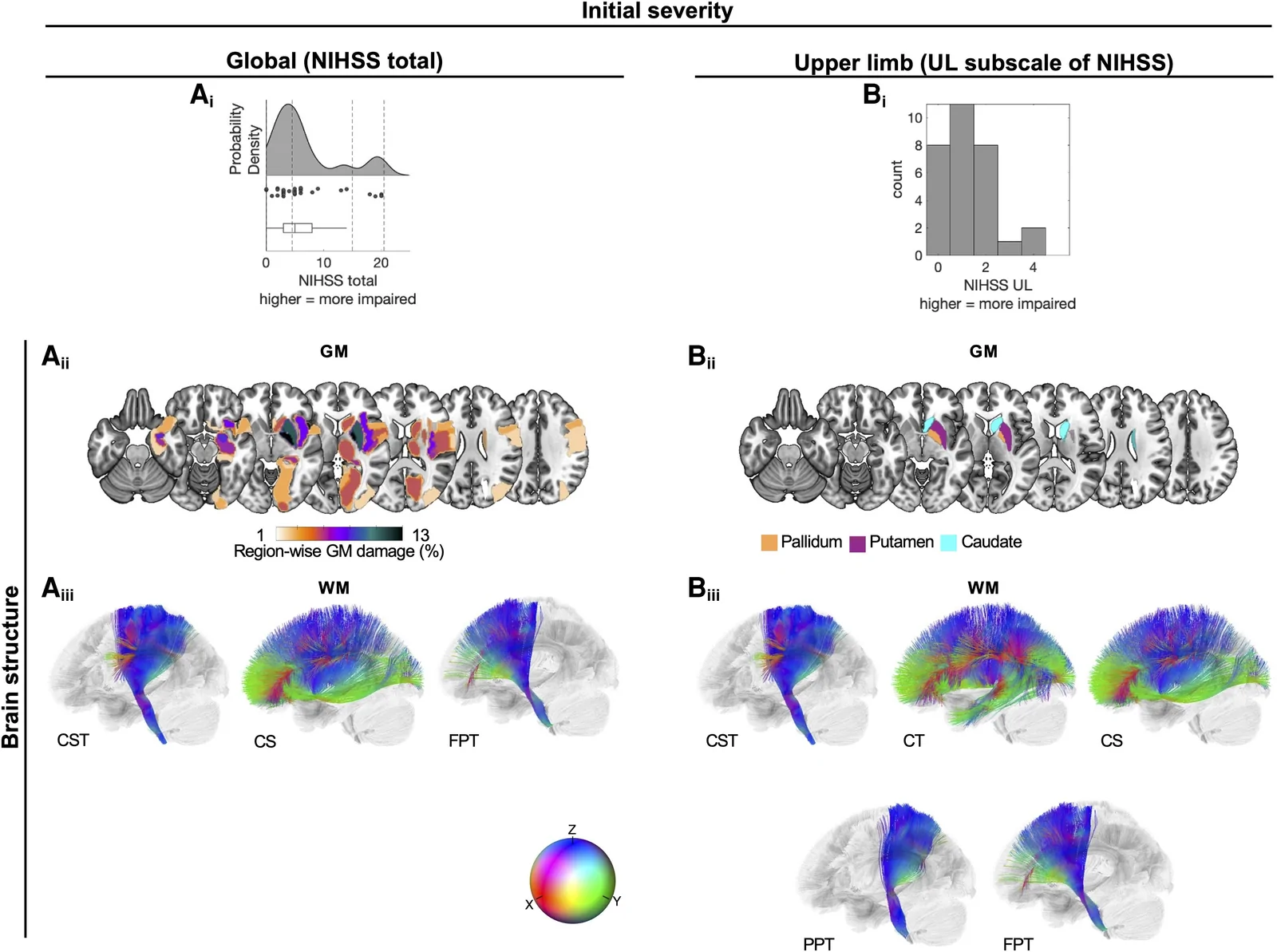
**Relationship between brain structure and global and UL-specific initial severity.** (**Ai**) Distribution of global initial severity (i.e. NIHSS total at hospital admission). Data are shown as probability function, individual subjects, and boxplot [median, interquartile range (25th–75th percentiles), whiskers extending to ±1.5× IQR]. Dashed lines represent different severity levels (minor stroke = 1–4, moderate stroke = 5–15, moderate to severe stroke = 16–20). (**Aii**) Global initial severity relates to total GM damage (*N* = 30). Illustrated is the Group region-wise map (same as Fig. 2B_i_, see Table 2 for the most damaged GM regions) of damage overlaid on an MNI template. Left hemispheric lesions were flipped. (**Aiii**) Global initial severity relates to greater WM damage of the following WM tracts (*N* = 30): corticospinal tract (CST), corticostriatal pathway (CS), and frontopontine tract (FPT, see Table 3 for the most damaged WM tracts). Tracts are colour-coded based on a standard red-green-blue (RGB) code applied to each (red for right-left, blue for dorsal-ventral, and green for anterior-posterior). (**Bi**) Distribution of UL-specific initial severity (i.e. UL subscale of NIHSS). Data are shown as probability function, individual subjects, and boxplot [median, interquartile range (25th–75th percentiles), whiskers extending to ±1.5× IQR]. (**Bii**) UL-specific severity relates to GM damage in the following GM regions (*N* = 30): pallidum (orange), putamen (magenta), caudate (cyan). (**Biii**) UL-specific initial severity relates to greater WM damage of the following WM tracts (*N* = 30): corticospinal tract (CST), corticothalamic pathway (CT), corticostriatal pathway (CS), parietopontine tract (PPT), and frontopontine tract (FPT). Tracts are colour-coded based on a standard red-green-blue (RGB) code applied to each (red for right-left, blue for dorsal-ventral, and green for anterior-posterior).

Regarding brain function, no significant relationships were found between global or UL-specific initial severity and resting state (any channel and any frequency) or β-power induced by passive movement (any channel and any time point) (*threshold*_cluster_forming_ = 3, *p*_cluster_threshold_ = 0.01, SI Fig. 2A, B).

### 6-month outcome while accounting for initial severity relates to brain function but not brain structure in acute stroke

We found no significant relationships between brain structure measures and global or UL-specific 6-month outcome (SIS) after controlling for initial severity (NIHSS, all *p*’s > 0.1).

Regarding brain function, greater β-ERD (i.e. more negative) in the contralateral (ipsilesional) hemisphere, induced by passive movement of the affected hand, was associated with both global and UL-specific 6-month outcome while accounting for initial severity (Table 4, Fig. 5Aii, Fig. 5Bii). This effect was specific to β-ERD induced by passive movement of the affected hand and not observed for the unaffected hand (*threshold*_cluster_forming_ = 3, *p*_cluster_threshold_ = 0.01, SI Fig. 3). For resting state activity, lower power in lower frequencies and higher power in higher frequencies were associated to both global and UL-specific 6-month outcome while accounting for initial severity (Table 4, Fig. 5Aiii, Fig. 5Biii).

**Figure 5:**
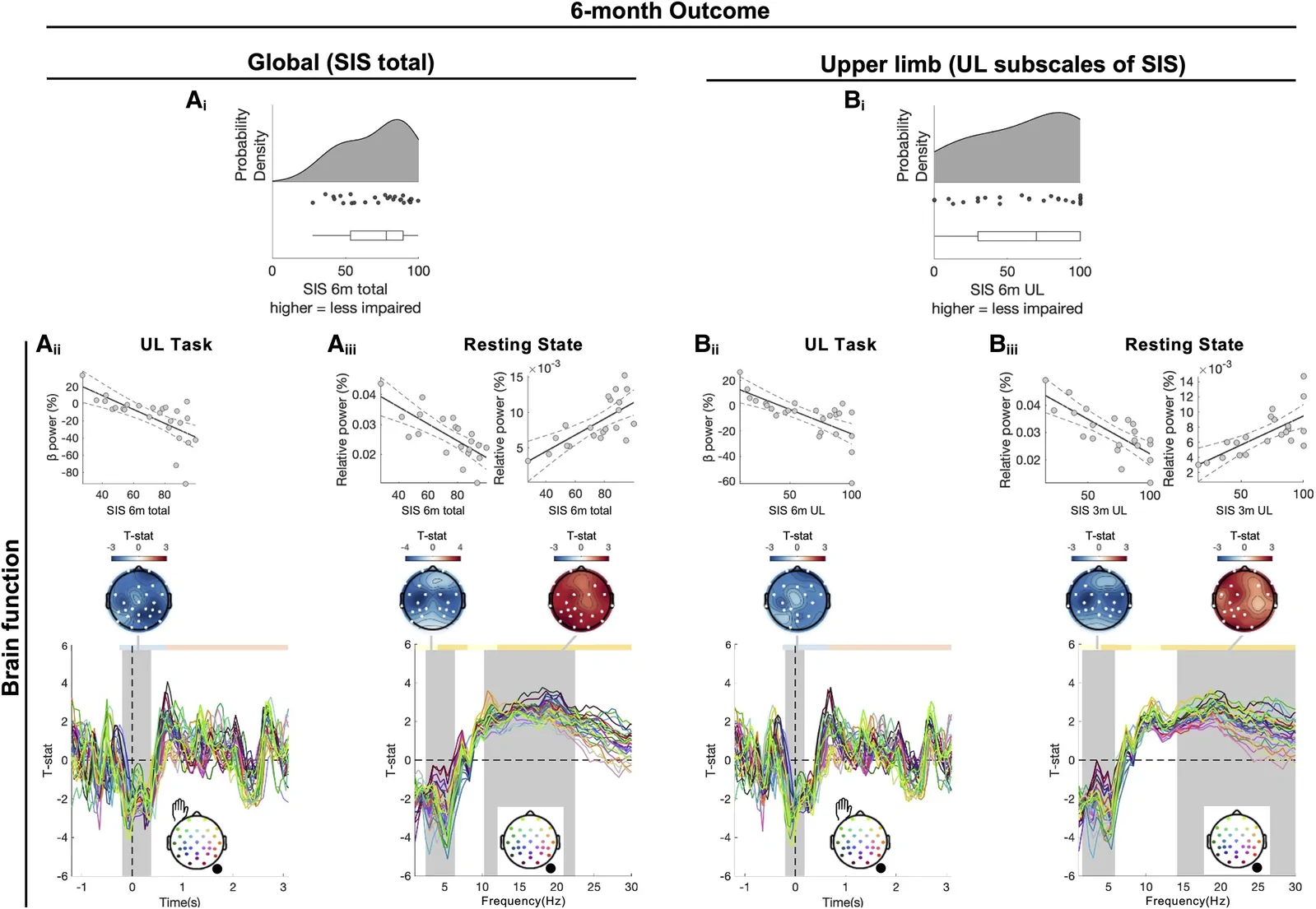
**Relationship between brain function and global and UL-specific 6-month outcome while accounting for initial severity.** (**Ai**) Distribution of global 6-month outcome (i.e. SIS total 6 months post-stroke). Data are shown as probability function, individual subjects, and boxplot [median, interquartile range (25th–75th percentiles), whiskers extending to ±1.5× IQR]. (**Aii**) (bottom) *t*-values as a function of time and space for the contrast correlating β-power during passive movement of the affected hand and SIS total, while accounting for NIHSS total (*N* = 27). The inlay illustrates the channel location colour coding, the lesioned hemisphere (black circle) and the hand that was moved (here affected hand). ERD and ERS time windows are highlighted in blue and red accordingly. Statistical significance is assessed by time × channel cluster permutation testing (*threshold*_cluster_forming_ = 3, *p*_cluster_threshold_ = 0.01). Significant times are indicated by the grey area. Significant channels are highlighted in white in the topography. (top) Visual representation of the data in the significant cluster, i.e. the correlation between β-power of the significant time × channel cluster and total SIS. (**Aiii**) (bottom) *t*-values as a function of frequency and space for the contrast correlating resting state power and SIS total, while accounting for NIHSS total (*N* = 21). The inlay topography illustrates the channel location colour coding, the lesioned hemisphere (black circle). δ-, θ-, α-, β-frequency bands are highlighted in yellow. Statistical significance is assessed by frequency × channel cluster permutation testing (*threshold*_cluster_forming_ = 3, *p*_cluster_threshold_ = 0.01). Significant frequencies are indicated by the grey area. Significant channels are highlighted in white in the topography. (top) Visual representation of the data in the significant cluster, i.e. the correlation between resting state power of the significant time × channel cluster and total SIS. (**Bi**) Distribution of UL-specific 6-month outcome (i.e. SIS total 6 months post-stroke). (**Bii**) Same as (Ai) for UL-specific 6-month outcome. (**Biii**) Same as (Aii) for UL-specific 6-month outcome.

**Table 4:** Cluster statistic for significant clusters in contrasts examining the relationship between brain function and global and UL-specific 6-month outcome while accounting for initial severity

Index	Comparison	No of subjects	*Rank*_cluster_	*T*_cluster___sum_	*T*_cluster_max_	*p*_cluster_	Time window	Frequency range	No of channels	*b* range	b mean
1	Global (SIS)—Passive Movement Task	27/30	1	−254.96	−4.12	0.030	−0.20–0.40 s	NA	26	−41.04–7.33	−13.58
2	Global (SIS)—Resting State	21/30	1	615.07	3.76	0.030	NA	10.25–22.46 Hz	23	7.30 × 10^−4^–2.40 × 10^−3^	9.00 × 10^−3^
3		21/30	2	−309.32	−5.12	0.039	NA	2.44–6.35 Hz	21	−1.39 × 10^−2^—−5.33 × 10^−4^	−5.4 × 10^−3^
4	UL subscale (SIS)—Passive Movement Task	27/30	1	−146.96	−4.44	0.042	−0.20–0.20 s	NA	19	−26.24–2.90	−9.75
5	UL subscale (SIS)—Resting State	21/30	1	432.05	3.62	0.040	NA	14.16–31.25	20	−2.26 × 10^−4^–1.90 × 10^−3^	3.00 × 10^−3^
6		21/30	2	−312.32	−5.07	0.042	NA	1.46–5.86	22	−2.76 × 10^−2^—−5.90 × 10^−3^	−1.20 × 10^−3^

For all contrasts in this table applies: *threshold*_cluster_forming_ = 3, *p*_cluster_threshold_ = 0.01. Note the different N of subjects across comparisons, is because missing data in resting state recording, SIS, NISS. NIHSS is used as covariate in this analysis, to analyse 6-month outcome when accounting for initial severity. *b* refers to regression coefficient.

### Combining clinical measures, brain structure, and brain function to model 6-month outcome

While the previous analysis examined the direct relationship between either brain structure or brain function and initial severity or 6-month outcome, we next aimed to assess how relevant measures of brain structure and function contribute to model 6-month outcome. First, linear regression was used to test whether initial severity models 6-month outcome. Global initial severity explained 42.9% of the variance in global 6-month outcome [*RMSE* = 57.1%, *F*(1,25) = 18.79, *P* < 0.001, see Table 5 for coefficients], and the UL-specific initial severity explained 30.4% of the variance in UL-specific 6-month outcome [*RMSE* = 58.84, *F*(1,25) = 10.92, *P* = 0.003, see Table 6 for coefficients]. Next, adding brain structure (i.e. CST damage) as an independent variable improved the model [global 6-month outcome: *ΔR*^2^ = 16.1%, *ΔF*(1,24) = 9.46, *P* = 0.005 relative to the initial model, see Table 5 for coefficients; UL-specific 6-month outcome: *ΔR*^2^ = 16.4%, *ΔF*(1,24) = 7.41, *P* = 0.012, see Table 6 for coefficients]. Adding brain function (i.e. task-related β-ERD) to the original model also improved the model [global 6-month outcome: *ΔR*^2^ = 31.9%, *ΔF*(1,24) = 30.33, *P* < 0.001 relative to the initial model, see Table 5 for coefficients; UL- specific 6-month outcome: *ΔR*^2^ = 37.6%, *ΔF*(1,24) = 28.18, *P* < 0.001, see Table 6 for coefficients]. Finally, a model combining initial severity, brain structure, and brain function provided a significantly better fit than models including initial severity with brain structure or brain function alone. Relative to the initial severity and brain structure model, adding brain function significantly increased explained variance [global 6-month outcome: *R*^2^ = 80.0, *ΔR*^2^ = 20.9, *F*change(1,23) = 23.98, *P* < 0.001, see Table 5 for coefficients; UL- specific 6-month outcome: *R*^2^ = 73.6, *ΔR*^2^ = 26.8%, *F*change(1,23) = 23.40, *P* < 0.001, see Table 6 for coefficients]. Relative to the initial severity and brain function model, adding brain structure also significantly increased explained variance [global 6-month outcome: *ΔR*^2^ = 5.2, *F*change(1,23) = 5.94, *P* = 0.023, see Table 5 for coefficients; UL- specific 6-month outcome: *ΔR*^2^ = 5.7, *F*change(1,23) = 4.94, *P* = 0.036, see Table 6 for coefficients]. The final model was confirmed via forward stepwise multivariate regression. For both global and UL-specific 6-month outcome, the full model—comprising initial severity, brain structure, and brain function—was retained. Sensitivity analyses demonstrated that the associations between initial severity, brain structure, and brain function and 6-month outcome remained significant after individually adjusting for age, lesion side, and time post-stroke. Inclusion of these covariates resulted in only small changes in model fit (all *ΔR*^2^ < 3) and standardized regression coefficients (all absolute *Δβ* < 10%), indicating that the observed relationships were robust to these factors.

**Table 5:** The coefficient table of the regression analysis for global 6-month outcome

Index	Variable	Unstand. coeff.		Sta. coeff.	*t*	*P*	95% CI	
		*β*	SE	*β*			Lower	Upper
1	NIHSS	5.48	1.26	1.58	4.34	<0.001	2.88	9.08
2	NIHSS	2.61	1.44	0.75	1.82	0.081	−0.35	5.57
	CST	0.65	0.21	1.19	3.08	0.005	0.21	1.09
3	NIHSS	4.48	0.87	1.36	5.44	<0.001	2.94	6.52
	β activity	−1.42	0.26	−1.72	−5.01	<0.001	−1.96	−0.89
4	NIHSS	3.11	1.03	0.90	3.02	0.006	0.98	5.24
	CST	0.39	0.16	0.71	2.44	0.023	0.06	0.72
	β activity	−1.22	0.25	−1.48	−4.90	<0.001	−1.74	−0.71

**Table 6:** The coefficient table of the regression analysis for UL-specific 6-month outcome

Index	Variable	Unstand. coeff.		Sta. coeff.	*t*	*P*	95% CI	
		*β*	SE	*β*			Lower	Upper
1	NIHSS	22.91	6.93	0.87	3.31	0.003	8.63	37.19
2	NIHSS	6.94	8.53	0.26	0.81	0.42	−10.66	24.53
	CST	0.64	0.24	0.83	2.72	0.012	0.156	1.13
3	NIHSS	21.15	4.81	0.80	4.40	<0.001	11.22	31.08
	β activity	−2.32	0.44	−1.31	−5.31	<0.001	−2.91	−1.42
4	NIHSS	11.56	6.21	0.44	1.86	0.075	−1.28	24.40
	CST	0.39	0.18	0.51	2.22	0.036	0.03	0.76
	β activity	−2.05	0.42	−1.15	−4.84	<0.001	−2.93	−1.17

## Discussion

Here we asked how brain structure and function in acute stroke using standard imaging, clinical measures, and bedside EEG, relate to global and UL initial severity and 6-month outcome while accounting for initial severity. In our patient cohort, initial severity was linked to structural damage, but not to functional measures. In contrast, 6-month outcome while accounting for initial severity was associated with brain function, but not structural damage. Nevertheless, the best model of 6-month outcome came from combining initial severity, brain structure, and function, highlighting their complementary value.

### Brain structure relates to initial severity but not 6-month outcome while accounting for initial severity

Here, we found that global initial severity was associated with lesion volume, total GM damage, and damage to specific WM tracts, including the CST, corticostriatal pathway, and frontopontine tract. No associations were observed with GM damage in individual regions. In contrast, UL-specific severity was linked to GM damage in the pallidum, putamen, and caudate, as well as damage to the CST, frontopontine, parietopontine, corticothalamic, and corticostriatal pathways.

The CST’s role in motor function explains its consistent association with both global and UL-specific severity. This aligns with prior work linking CST integrity (fractional anisotropy, lesion load, or fibre count) to acute motor function/impairment (ARAT,^5,6^ BB,^7^ FM-UE,^8,9^ NHPT,^7^ NIHSS,^6,7,9^ grip strength^7^), and to somatosensory deficits.^60^ These relationships often persist beyond the acute stage (BB,^61^ grip strength,^62^ FM-UE^61,63^). In this study, in addition to the CST, damage to the frontopontine and parietopontine tracts was also linked to initial severity. These tracts share anatomical proximity with the CST and are part of the corticopontine system.^64^ The observed associations with the corticothalamic and corticostriatal pathways implicate the cortico-basal ganglia-thalamocortical circuits,^65^ which support motor planning and reinforcement learning.^66^ GM damage in the basal ganglia, in line with,^67^ may reflect disruption of these circuits. Initial severity was also associated with damage to the corticothalamic and corticostriatal pathways. These form part of the cortico-basal ganglia-thalamocortical circuits^65^ involved in motor planning and reinforcement learning.^66^ GM damage to the pallidum, putamen, and caudate, consistent with,^67^ may reflect disruption of these pathways.

In the present patient cohort, we did not observe significant associations between structural brain damage and 6-month outcome when accounting for initial severity. Although prior studies report associations between CST integrity and recovery (ARAT,^5,15^ FM-UE,^16^ NIHSS^15^), many studies do not control for initial severity—a key factor closely tied to structural damage—making it difficult to distinguish true recovery-related effects from baseline impairment. Moreover, these findings should be considered in the context of the study’s limitations outlined below.

### Brain function relates to 6-month outcome while accounting for initial severity, but not initial severity

#### Brain function during task relates to 6-month outcome while accounting for initial severity

While brain structure provides valuable insights, it is not sufficient on its own to model recovery; incorporating measures of brain function is important for better understanding of recovery trajectories. Consistent with prior studies, we observed significant differences in sensorimotor β-activity between stroke survivors in the acute stage post-stroke and HC when probing the sensorimotor system.^34-36^ Unlike previous studies that focused exclusively on post-movement β-ERS and on the affected UL, limiting their ability to assess the limb- and component-specificity of post-stroke changes, we analysed the full β-ERD–ERS complex for both the affected and unaffected ULs. Compared with HC, β-ERD was reduced (less negative) only during passive movement of the affected UL, while β-ERS was reduced (less positive) during movement of both ULs. No association was observed between β-activity and initial motor severity, but β-ERD during passive movement of the affected UL, and not the unaffected UL, was linked to 6-month outcome while accounting for initial severity. The selective reduction of β-ERD (less negative) during passive movement of the affected UL, and its association with 6-month outcome while accounting for initial severity, suggests a limb-specific and functionally meaningful alteration, whereas the β-ERS points to a more global, non-specific change in post-stroke cortical processing. This is further supported by the observation that β-ERS, for both the affected and unaffected UL, but not β-ERD, shows significant correlations with global structural metrics, such as lesion volume and total GM damage. Mechanistically, β-ERD reflects sensorimotor cortex engagement during movement, linking it to limb-specific motor output and recovery. In contrast, post-movement β-ERS reflects broader processes, such as resetting cortical excitability and maintaining sensorimotor stability, making it more susceptible to global alterations after stroke.

Earlier MEG studies using tactile stimulation reported stronger β-ERS (more positive) correlating with lower initial severity and better recovery (BB,^35,36^ NHPT^13,34,35,36^). Our study, using passive movement and bedside EEG, found a link between β-ERD and 6-month outcome while accounting for initial severity. Differences in findings may stem from outcome measures (NIHSS versus NHPT/BB) and stimulation type tactile stimulation^34,36^ versus passive movement.^35,36^ Passive movement is known to elicit stronger β-ERS (more positive) and comparable β-ERD in HC.^68^ Additionally, while prior studies used MEG, we employed high-quality EEG, which, despite lower signal-to-noise ratio, shows strong cross-modality β-feature correlations.^69^ Sample variability (stroke type, lesion site, initial severity, age, alertness) may also explain inter-study differences, especially given small acute stroke cohorts.^23,70^ Notably, the relatively limited extent of GM damage suggests that EEG recordings in this cohort are less likely to be substantially confounded by widespread cortical tissue loss. Overall, early post-stroke β-activity may serve as a functional marker of motor recovery, though the distinct roles of β-ERD and ERS require further exploration.

#### Brain function during rest relates to 6-month outcome while accounting for initial severity

We found no significant differences in resting-state spectral power (1–30 Hz) between acute stroke survivors and HC across EEG sensors. This aligns with MEG studies reporting no differences in β-peak amplitude at rest between hemispheres, time points, or groups.^7,34-36^ Similarly,^71^ found no group or hemispheric differences in α/β-power under eyes-open or eyes-closed conditions. In contrast, Wu and colleagues showed that δ/β-power in specific clusters predicted initial severity (NIHSS) in 25 stroke survivors 3 h—12 days post-stroke.^72^

Regarding recovery, in our study, lower resting δ/θ-power and higher α/β-power were associated with better global and UL 6-month outcome while accounting for initial severity. These results align with previous findings linking elevated low-frequency power in acute stroke with poorer outcomes (FM-UE,^73^ NHPT,^71^ NIHSS^71,74^). Such increases may reflect cortical deafferentation,^75,76^ perilesional pathological activity,^77^ or axonal sprouting.^78^ Additionally, our findings match results from a randomized trial where increased β-power post-anodal tDCS was linked to improved lower limb function (FM-UE).^79^ In contrast,^71^ found no link between α/β-power and outcome (NHPT, NIHSS), though they did not adjust for initial severity, possibly explaining the inconsistency.

We and others report that recovery-related power changes often showed bilateral topographies. Potential mechanisms for contralesional effects include mass effects,^80,81^ transcallosal diaschisis,^82^ altered inhibition and excitability,^83-85^ and interhemispheric imbalance.^86^

### Integrating clinical, structural, and functional measures to model 6-month outcome post-stroke

After examining the direct associations of brain structure and function with initial severity and 6-month outcome, we next assessed how these measures together model recovery trajectories. Models that combined initial severity, brain structure, and brain function explained global and UL-specific 6-month outcome better than any single measure alone. In the correlation analysis brain structure did not show a significant association with 6-month outcome, however, in regression models that included initial severity and brain function, adding structural information still explained additional unique variance, indicating it provides complementary information that enhances the modelling of recovery trajectories. Notably, adding brain functional measures produced a larger improvement in model fit than adding structural measures, which may reflect the close relationship between structural damage and initial severity, reducing the additional predictive contribution of structure. While demographic factors (e.g. age, sex)^87-89^ and clinical characteristics (e.g. stroke type, treatment, lesioned hemisphere)^90,91^ are known to influence stroke recovery, the stepwise regression analysis demonstrates that markers of brain function and brain structure derived from EEG and clinical MRI provide additional, complementary information beyond routine clinical measures, underscoring the importance of integrating these modalities in future adequately powered studies. The added value of combining brain function and structure has previously been demonstrated in predicting impairment (acute stage,^72^ chronic stage^92,93^), recovery,^13,94^ and therapy response in the subacute stage.^95^ These findings suggest that stroke-related changes in brain function are not merely a consequence of structural damage; if they were, brain function would not account for variance in recovery beyond that explained by brain structure alone.

### Limitations and future directions

There are a number of limitations with the present study, which are hopefully overcome by future research. While the sample size reflects the current landscape of research in acute stroke^70^ it limits the conclusions we can draw from these results. The underrepresentation of female participants in our cohort is a limitation, consistent with trends commonly observed in acute stroke research.^70^ Despite the challenges of acute stroke research more studies with larger, more diverse cohorts are needed to understand how stroke type, lesion location, and patient factors influence recovery. Our study focused on clinical tools (CT, MRI), standard assessments (e.g. NIHSS, although the coarse grading of the UL-NIHSS item may limit sensitivity to subtle differences), and bedside-compatible EEG, while 6-month outcome was measured with the SIS—a validated, reliable, and remotely administered patient-reported outcome capturing disability and quality of life. Thus, the structural measures here combined GM injury and disconnectome estimates, which provide a validated proxy for WM disconnection,^96^ derived from routine clinical MRI rather than individual DTI at research field strengths. Additional measures of brain function, such as motor-evoked potentials from transcranial magnetic stimulation or functional network activity via fMRI, could provide further insight. Similarly, incorporating detailed motor and sensory assessments could further improve characterization of initial severity and outcome. However, the pragmatic nature of our data still allows us to draw useful insights about the nature of the relationship between brain structure/function and initial severity/outcome. Developing standardized protocols that combine accessible bedside measures with advanced imaging can enhance individualized prognosis and support targeted therapies. These advances hold promise for improving stroke recovery outcomes and personalizing patient care.

## Supplementary Material

fcag355_Supplementary_Data

## Data Availability

All data produced in the present study are available upon reasonable request to the authors. The code for the analysis is available at: https://github.com/cathazi/Zich_2026_AcuteStroke.git.
