# Supplementary material for: Stroke recovery beyond initial severity: the complementary roles of brain structure and brain function in acute stroke
Source: Brain Commun. 2026 Sep 16;8(5):fcag355. doi: 10.1093/braincomms/fcag355 (PMC13621915; doi:10.1093/braincomms/fcag355)
Supplement: Supplementary file 1 [file fcag355_supplementary_data.docx]

**Supplemental Information**

**Stroke recovery beyond initial severity: the complementary roles of brain structure and brain function in acute stroke**

Catharina Zich, Lydia C Mardell, Andrew J Quinn, Sven Bestmann, Nick S Ward

**SI Fig. 1. β-power related to passive movement of the unaffected hand relative to healthy controls**

**Ai)** β-power during passive movement for healthy controls of the dominant hand. Mean power estimates as a function of time and space. The inlay illustrates the channel location colour coding and the hand that was moved (dominant hand). ERD and ERS time windows are highlighted in blue and red accordingly. Topographies show the spatial pattern of β-power averaged over ERD and ERS time windows.

**Aii)** Same as Ai) for passive movement of the unaffected hand in stroke survivors. The inlay illustrates the channel location colour coding, the lesioned hemisphere (black circle) and the hand that was moved (here unaffected hand).

**Aiii)** t-values as a function of time and space for the contrast between healthy controls (N = 26) and acute stroke survivors (N = 30). Statistical significance is assessed by time x channel cluster permutation testing (*threshold_cluster_forming_* = 3, *p_cluster_threshold_* = 0.01). Significant times are indicated in by the grey area. Significant channels are highlighted in white in the topography.

**SI Table 1.** **Cluster statistic for significant clusters in contrasts examining the difference between β-power induced by passive movement of the unaffected hand in stroke survivors and healthy controls (random hand).** For all contrasts in this table applies: *threshold_cluster_forming_* = 3.5, *p_cluster_threshold_* = 0.01.

| Index | Comparison | *rank_cluster_* | *T statisitc* | | *p_cluster_* | Time window | N of channels | β-ERS difference | |
| --- | --- | --- | --- | --- | --- | --- | --- | --- | --- |
|  |  |  | *T_cluster___sum_* | *T_cluster_max_* |  |  |  | range | mean |
| 1 | Healthy Controls > Acute | 1 | 283.33 | 5.07 | < 0.001 | 2.05 – 2.65 s | 19 | 4.26 – 50.76 | 32.90 |
| 2 |  | 2 | 192.81 | 4.12 | < 0.001 | 1.00 – 1.35 s | 15 | 26.28 – 62.83 | 48.04 |
| 3 |  | 3 | 72.09 | 4.40 | < 0.001 | 1.55 – 1.85 s | 7 | 23.22 – 53.71 | 37.79 |
| 4 |  | 4 | 66.46 | 3.92 | < 0.001 | 1.60 – 1.95 s | 5 | 19.05 – 47.79 | 37.12 |

**SI Fig. 2. Relationship between brain function and global and UL-specific initial severity**

**Ai)** Global initial severity (i.e., NIHSS total from hospital admission) as probability function, individual subjects, and boxplot (median, interquartile range [25th–75th percentiles], whiskers extending to ±1.5× IQR). Dashed lines represent different severity levels (minor stroke = 1-4, moderate stroke = 5-15, moderate to severe stroke = 16-20).

**Aii)** t-values as a function of time and space for the contrast correlating β-power during passive movement of the affected hand and NIHSS total (N = 30). The inlay illustrates the channel location colour coding, the lesioned hemisphere (black circle) and the hand that was moved (here affected hand). ERD and ERS time windows are highlighted in blue and red accordingly. Statistical significance is assessed by time x channel cluster permutation testing (*threshold_cluster_forming_* = 3, *p_cluster_threshold_* = 0.01). No significant cluster were found.

**Aiii)** t-values as a function of frequency and space for the contrast correlating resting state power and NIHSS (N = 21). The inlay topography illustrates the channel location colour coding, the lesioned hemisphere (black circle). δ, θ, α, β- frequency bands are highlighted in yellow. Statistical significance is assessed by frequency x channel cluster permutation testing (*threshold_cluster_forming_* = 3, *p_cluster_threshold_* = 0.01). No significant cluster were found.

**Bi)** Distribution of UL-specific initial severity (i.e., UL subscale of NIHSS).

**Bii)** Same as Ai) for UL-specific initial severity.

**Biii)** Same as Aii) for UL-specific initial severity.

**SI Fig. 3. Relationship between β-power induced by passive movement of the unaffected hand and global and UL-specific 6-month outcome while accounting for initial severity**

**Ai)** Global 6-month outcome (i.e., SIS total three months post stroke) as probability function, individual subjects, and boxplot (median, interquartile range [25th–75th percentiles], whiskers extending to ±1.5× IQR).

**Aii)** t-values as a function of time and space for the contrast correlating β-power during passive movement of the unaffected hand and SIS total, while accounting for NIHSS total (N = 27). The inlay illustrates the channel location colour coding, the lesioned hemisphere (black circle) and the hand that was moved (here unaffected hand). ERD and ERS time windows are highlighted in blue and red accordingly. Statistical significance is assessed by time x channel cluster permutation testing (*threshold_cluster_forming_* = 3, *p_cluster_threshold_* = 0.01). No significant cluster were found.

**Bi)** UL-specific 6-month outcome (i.e., SIS total three months post stroke) as probability function, individual subjects, and boxplot (median, interquartile range [25th–75th percentiles], whiskers extending to ±1.5× IQR).

**Bii)** Same as Ai) for UL-specific 6-month outcome.
